# *In vivo* and *in vitro* studies of Cry5B and nicotinic acetylcholine receptor agonist anthelmintics reveal a powerful and unique combination therapy against intestinal nematode parasites

**DOI:** 10.1371/journal.pntd.0006506

**Published:** 2018-05-18

**Authors:** Yan Hu, Melanie Miller, Bo Zhang, Thanh-Thanh Nguyen, Martin K. Nielsen, Raffi V. Aroian

**Affiliations:** 1 Program in Molecular Medicine, University of Massachusetts Medical School, Worcester, MA, United States of America; 2 Division of Biological Sciences, University of California, San Diego, La Jolla, CA, United States of America; 3 Department of Quantitative Health Sciences, University of Massachusetts Medical School, Worcester, MA, United States of America; 4 Department of Veterinary Science, University of Kentucky, Lexington, KY, United States of America; University of Pennsylvania, UNITED STATES

## Abstract

**Background:**

The soil-transmitted nematodes (STNs) or helminths (hookworms, whipworms, large roundworms) infect the intestines of ~1.5 billion of the poorest peoples and are leading causes of morbidity worldwide. Only one class of anthelmintic or anti-nematode drugs, the benzimidazoles, is currently used in mass drug administrations, which is a dangerous situation. New anti-nematode drugs are urgently needed. *Bacillus thuringiensis* crystal protein Cry5B is a powerful, promising new candidate. Drug combinations, when properly made, are ideal for treating infectious diseases. Although there are some clinical trials using drug combinations against STNs, little quantitative and systemic work has been performed to define the characteristics of these combinations *in vivo*.

**Methodology/Principal findings:**

Working with the hookworm *Ancylostoma ceylanicum*-hamster infection system, we establish a laboratory paradigm for studying anti-nematode combinations *in vivo* using Cry5B and the nicotinic acetylcholine receptor (nAChR) agonists tribendimidine and pyrantel pamoate. We demonstrate that Cry5B strongly synergizes *in vivo* with both tribendimidine and pyrantel at specific dose ratios against hookworm infections. For example, whereas 1 mg/kg Cry5B and 1 mg/kg tribendimidine individually resulted in only a 0%-6% reduction in hookworm burdens, the combination of the two resulted in a 41% reduction (P = 0.020). Furthermore, when mixed at synergistic ratios, these combinations eradicate hookworm infections at doses where the individual doses do not. Using cyathostomin nematode parasites of horses, we find based on inhibitory concentration 50% values that a strongylid parasite population doubly resistant to nAChR agonists and benzimidazoles is more susceptible or “hypersusceptible” to Cry5B than a cyathostomin population not resistant to nAChR agonists, consistent with previous *Caenhorhabditis elegans* results.

**Conclusions/Significance:**

Our study provides a powerful means by which anthelmintic combination therapies can be examined *in vivo* in the laboratory. In addition, we demonstrate that Cry5B and nAChR agonists have excellent combinatorial properties—Cry5B combined with nAChR agonists gives rise to potent cures that are predicted to be recalcitrant to the development of parasite resistance. These drug combinations highlight bright spots in new anthelmintic development for human and veterinary animal intestinal nematode infections.

## Author summary

Intestinal nematodes are roundworm parasites of humans and animals, causing significant morbidity in both. In humans, these parasites are leading causes of morbidity in children, *e*.*g*., causing growth stunting, cognitive impairment, and malnutrition. Few drugs are used to treat these parasites in humans and animals and there is increasing evidence that the drugs are losing efficacy and/or have low efficacy. Infectious diseases are best treated with drug combinations and not single drugs. However, there has been little work to characterize in detail how various anti-nematode drugs combine. Here we establish a new laboratory model to study anti-nematode drug combinations using the human hookworm *Ancylostoma ceylanicum* infection in hamsters. We show that two classes of anti-nematode drugs, Cry5B and the nicotinic acetylcholine receptor agonists tribendimidine and pyrantel, combine (synergize) in a way that is more powerful at specific drug ratios than predicted from their individual impacts. Furthermore, when combined at these ratios, these combinations completely eliminated parasites at doses where normally neither drug has that effect. Horse parasites resistant to pyrantel also appear to be hypersensitive (more sensitive than wild-type parasites) to Cry5B. These characteristics predict that combinations of Cry5B with tribendimidine or pyrantel will be extremely effective therapeutically and relatively recalcitrant to the development of parasite resistance.

## Introduction

Soil-transmitted helminth or soil-transmitted nematode (STN) infections are caused by different species of intestinal parasitic nematodes, mainly *Ascaris lumbricoides*, the hookworms *Necator americanus*, *Ancylostoma duodenale* and *Ancylostoma ceylanicum*, and *Trichuris trichiura[1]*. STNs are the most prevalent parasites of humans on earth, with approximately 1.5 billion people, or 20% of the world’s population, infected with at least one of these parasites[2]. STN infections can cause severe morbidity especially in children, including growth stunting, intellectual impairment, cognitive and educational deficits, malnutrition, and iron deficiency anemia; they also have significant impacts on adults including complications with pregnancy, impaired worker productivity and productive capacity, and weak adults[1,3]. Intestinal parasitic nematodes are also the most common parasites of farm and companion animals worldwide and are significant problems in veterinary medicine. In horses, for example, strongylid parasites are considered ubiquitous and known as a common cause of gastrointestinal disease [4].

Drug treatment using anti-nematode drugs (anthelmintics) is the current strategy to control morbidity associated with STN infections but perilously relies heavily on a single class of drug, the benzimidazoles (BZs, namely albendazole, mebendazole) [5,6]. Unfortunately, the current efficacy of BZs is in an alarming situation: 1) only albendazole has good efficacy against hookworm infection and neither is highly effective against the whipworm *T*. *trichiura [7]*; 2) not all mass drug administrations are achieving encouraging results [8,9]; and 3) there are clear examples of low BZ efficacy against both hookworms and Ascaris, even with albendazole [10–13]. Relying on a single class of drug for parasitic nematode diseases is clearly a recipe for disaster, as has been commonly seen in veterinary medicine where resistance to single drug classes is rampant [14]. A second approved class of anti-STN (anthelmintic) drugs is the nicotinic acetylcholine receptor (nAChR) agonists, namely pyrantel and levamisole. However, these have much lower efficacy than the BZs and thus are typically not used [5,6]. Tribendimidine, a newer nAChR agonist that is not yet on the WHO approved drug list, has better efficacy (similar to albendazole) and is moving forward for clinical approval [15–19]. The same classes of drugs are commonly used against intestinal nematodes in veterinary medicine, where widespread resistance of many parasites in many animal hosts is common and medically problematical [14,20].

Drug combinations to delay/prevent resistance are the mainstay of the “big three” global infectious diseases: HIV, TB, and malaria [21]. It is now only with drug combinations that there is effective therapy against these infectious disease agents, which would otherwise rapidly develop resistance to monotherapies. Currently, drug combinations are routinely used to target filarial nematode diseases [22] but not STN diseases. There is clearly an increased interest in clinical studies with anti-STN drugs in which treatment trials are compared with defined doses of various drugs singly and in combination, often, but not always, with the goal of improving efficacy against whipworms [16,23–27]. However, because of the limitations of carrying out clinical studies with humans, it is difficult to carry out detailed studies regarding optimization of combinations (*e*.*g*., studying effects of various ratios of drug combinations or studying effects of resistant alleles on drug efficacy) with regards to efficacy and long-term resistance management. *In vivo* animal models of STNs can be used to address these limitations.

Cry5B is a new class of anti-nematode compound with *in vivo* efficacy against hookworms and *Ascaris [28–31]*. Previous studies using the laboratory free-living nematode *Caenorhabditis elegans [32]* have demonstrated that Cry5B has two ideal anti-nematode combinatorial characteristics with nAChR agonists levamisole, pyrantel, and tribendimidine: 1) Cry5B synergizes with nAChR agonists to kill and intoxicate *C*. *elegans*; and 2) *C*. *elegans* resistant to nAChR agonists are hypersusceptible or hypersensitive to Cry5B. Here, we test these combinatorial results using parasitic nematodes, looking for synergy with hookworms *in vivo* and hypersensitivity (hypersusceptibility) with horse small strongylid parasites *in vitro*. For these studies, we develop a new system for studying *in vivo* anti-nematode combination therapies with Cry5B and nAChR agonists, with promising results. This methodology also allows for optimization of drug ratios that can be widely applied to any anti-nematode therapy and that can provide major advances in how to best combine anti-nematode therapies for human STN and veterinary medicine use.

## Materials and methods

### Ethics statement

Hamsters and horses were provided with food and water (ad libitum). All animal experiments were carried out under protocols approved by either the University of California, San Diego (UCSD; S09067), University of Massachusetts Medical School (UMMS; A-2483) or University of Kentucky (UK; 2012–1046) Institutional Animal Care and Use Committees (IACUC). All housing and care of laboratory animals used in this study conform to the National Institutes of Health (NIH) Guide for the Care and Use of Laboratory Animals in Research (see 18-F22) and all requirements and all regulations issued by the United States Department of Agriculture (USDA), including regulations implementing the Animal Welfare Act (P.L. 89–544) as amended (see 18-F23). Euthanasia was performed by CO2 asphyxiation, followed by bilateral pneumothorax.

### Nematode maintenance

Hookworms *A*. *ceylanicum* were maintained in golden Syrian hamsters [29,30,33,34]. The cyathostomins in this study were maintained naturally in two different herds. The pyrantel-susceptible cyathostomin eggs were collected from the feces of an equine herd kept without deworming since 1979 [35]; eggs of anthelmintic resistant cyathostomins (Population S) were collected from the feces of a herd harboring cyathostomins that are doubly resistant to pyrantel and benzimidazoles [36].

### Reagents and drugs

HD1 Cry5B spore crystal lysate (SCL) and HD1 spore lysate (SL) for *in vivo* studies were produced and the concentration of Cry5B protein in HD1-Cry5B SCL was determined as previously described [37]. Tribendimidine was kindly provided by Dr. Shu-Hua Xiao at the Chinese Centers for Disease Control and Prevention. Pyrantel pamoate was purchased from Sigma (Cat# P6210-5G). On the day of use, SL and Cry5B SCL aliquots, tribendimidine powder and pyrantel pamoate powder were resuspended in distilled water right before *in vivo* treatment. Cry5B SCL suspension was kept on ice until gavage. When combinations were used, the drugs were given together in a single oral gavage. SL was used as the control for the Cry5B dose-response experiment, but water was used as the control for other experiments. Repeated studies in our lab have shown that SL alone has no effect on parasite burden of fecal egg counts relative to water (manuscript in preparation). Purified Cry5B for cyathostomin developmental inhibition assays was produced as described before [30,38] and dissolved in 20 mM HEPES (pH8.0) right before setting up the assays.

### *In vivo* curative studies with *A*. *ceylanicum* infections in hamsters

Male hamsters were infected with standard protocol as described before [29,30,33,34]. Males were used because we find they are much more susceptible to hookworm infection than females. On day 17 post-inoculation (P.I.), an overnight fecal sample was collected from each infected hamster. The number of eggs present was counted using the modified McMaster technique and the hamsters were grouped to ensure that the hamsters in each treatment group had roughly equivalent infection levels [29]. On day 18 P.I., hamsters were individually weighed and given the relevant treatment *per os* based on body weight. Anthelmintics (or water control) were given in 0.4 mL volume in sterile double-distilled water (3–5 hamsters per group) through a blunt-ended gavage needle. On day 21 P.I., an overnight fecal sample was collected from each infected hamster. The hamsters were sacrificed on day 22 P.I., and parasite burdens and eggs per gram of feces were determined as described [29].

### *In vitro* developmental inhibition assays with cyathostomins

Horse fecal samples harboring wild-type or double resistant cyathostomins eggs were collected fresh in airtight plastic baggies, transported in coolers to the laboratory, packed, and shipped with ice packs to UMASS Medical School for egg isolation. Upon arrival, cyathostomin eggs were isolated using a modified nematode egg isolation protocol [39]. Larval development inhibition assays were set up in 96-well plates with purified Cry5B and pyrantel pamoate as described before for the *Caenorhabditis elegans* larval intoxication assay [40], except ~30 cyathostomin eggs were added to each well instead of *C*. *elegans* L1 larvae. Plates were incubated at 28° for 7 days. Each experiment included triplicate wells at each dose of Cry5B or pyrantel, and the experiment was performed three times independently.

### Statistical and synergy analyses

All the *in vivo* and *in vitro* data were plotted with Prism 7 (GraphPad Software, California, USA) and numerically presented in Tables 1, 2, 3, S1, and S2. The 95% confidence limits (given in S3 Table) and percentage reductions of the *in vivo* experiments shown in tables were calculated with Prism 7 and Microsoft Excel, respectively. We ran Shapiro-Wilk to test for normality of the data, and the data generally fit a normal distribution. However, to address any potential issues with non-normality, the *in vivo* data were analyzed by R statistical software [41] using a rank-based nonparametric multiple contrast test procedure by Konietschke and Pauly [42]. These are the values reported in the text. The *in vivo* data in all figures was also analyzed by IBM SPSS Statistics v. 26 one-way ANOVA with one-tailed Dunnett’s post-test adjustment. Both sets of values are reported in the tables. For both analyses, each data point was compared relative to placebo control (water). The cyathostomin *in vitro* data statistical analyses (IC_50_ values and 95% confidence limit calculations) were carried out with R statistical software.

**Table 1:** *In vivo* data associated with experimental results in Fig 3.

Treatment	Hookworm burden (% reduction) ^a^	P ^b^	P ^c^	Fecal egg counts (% reduction) ^d^	P ^b^	P ^c^
Control (water)	22.3	na	na	2072	na	na
0.33 mg/kg Cry5B	24.9 (-11.7)	0.99	0.99	1689 (18.5)	0.80	0.51
1 mg/kg Cry5B	24.3 (-9.0)	0.98	0.98	2150 (-3.8)	0.98	0.93
0.33 mg/kg TrBD	25.8 (-15.7)	0.99	1.00	1816 (12.4)	0.78	0.66
1 mg/kg TrBD	20.9 (6.3)	0.69	0.76	1534 (26.0)	0.66	0.31
0.33 mg/kg Cry5B + 0.33 mg/kg TrBD	15.9 (28.7)	0.13	0.12	1153 (44.4)	0.20	0.05
0.33 mg/kg Cry5B + 1 mg/kg TrBD	21.4 (4.0)	0.74	0.81	1316 (36.5)	0.38	0.12
1 mg/kg Cry5B + 0.33 mg/kg TrBD	17.1 (23.3)	0.21	0.23	1425 (31.2)	0.42	0.20
1 mg/kg Cry5B + 1 mg/kg TrBD	13.1 (41.3)	0.020	0.01	831 (60.0)	0.056	0.006

^a^ Average hookworm burdens (% reduction relative to water control)

^b^ P value relative to water control, non-parametric comparison. See Materials and Methods.

^c^ P value relative to water control, parametric comparison. See Materials and Methods.

^d^ Average fecal egg counts (% reduction relative to water control)

na: not applicable

TrBD = Tribendimidine

**Table 2:** *In vivo* data associated with experimental results in Fig 6.

Treatment	Hookworm burden (% reduction) ^a^	P ^b^	P ^c^	Fecal egg counts (% reduction) ^d^	P ^b^	P ^c^
Control (water)	37.3	na	na	3194	na	na
0.33 mg/kg PYR	33.5 (10.1)	0.74	0.75	2094 (34.4)	0.45	0.16
0.33 mg/kg Cry5B	33.3 (10.7)	0.79	0.74	2381 (25.5)	0.54	0.33
1 mg/kg Cry5B	32.8 (12.1)	0.78	0.71	2681 (16.1)	0.76	0.57
3 mg/kg Cry5B	39.8 (-6.7)	0.96	0.95	2556 (20.0)	0.71	0.47
9 mg/kg Cry5B	24.0 (35.7)	0.12	0.21	1575 (50.7)	0.21	0.025
0.33 mg/kg PYR + 0.33 mg/kg Cry5B	31.8 (14.8)	0.68	0.65	2325 (27.2)	0.56	0.29
0.33 mg/kg PYR + 1 mg/kg Cry5B	15.8 (57.6)	0.031	0.02	1850 (42.1)	0.24	0.070
0.33 mg/kg PYR + 3 mg/kg Cry5B	25.3 (32.2)	0.35	0.26	1900 (40.5)	0.34	0.084
0.33 mg/kg PYR + 9 mg/kg Cry5B	12.0 (67.8)	0.030	0.008	813 (74.6)	0.080	0.001

^a^ Average hookworm burdens (% reduction relative to water control)

^b^ P value relative to water control, non-parametric comparison. See Materials and Methods.

^c^ P value relative to water control, parametric comparison. See Materials and Methods.

^d^ Average fecal egg counts (% reduction relative to water control)

na: not applicable

PYR = Pyrantel

**Table 3:** Inhibitory concentration 50% (IC_50_) values in μg/mL with standard error for two cyathostomin lines treated with either pyrantel or Cry5B.

	Susceptible (Barn 10)	Pyrantel-resistant (Population S)	P value
IC_50_ on pyrantel	21.9 ± 5.2	65.8 ± 23.7	<0.001
IC_50_ on Cry5B	10.8 ± 1.4	7.7 ± 0.86	0.033

## Results

### Cry5B and tribendimidine are synergistic *in vivo*

Synergism is used to describe the situation where the addition of one agent apparently increases the effect of another so that the effect of a combination is greater than would be expected if additive. We had two goals for studying Cry5B and tribendimidine *in vivo*: 1) to determine if they are synergistic, as they are against the free-living nematode *C*. *elegans* “*in vitro*” [32]; and 2) to determine which ratio of Cry5B and tribendimidine may be more synergistic since the degree of synergy for these two anti-nematode compounds against *C*. *elegans* changes based upon the ratio of the drugs [32]. Methods for quantitating synergy include checkerboards, isobolograms, and the combination index method [43–46]. However, we found that these methods are not ideally suited for measuring synergy for STN infections *in vivo* because the high natural variation of worm burdens that occur in laboratory animals would require using large number of animals for such a study. Indeed, in contrast to other studies [47,48], a preliminary study we carried out using the combinatorial index method to study *in vivo* synergy with these drugs failed to give an interpretable result for these reasons. We therefore devised a different method for studying drug synergy against STNs *in vivo*.

We decided on an amalgam of approaches discussed in Poch and Holzmann [49,50] and Chou [51]. In the former papers the response to drug A over a range of doses is studied in combination with a fixed, but active, amount of drug B. Chou conversely discusses the concept of drug enhancement/potentiation/ augmentation, in which drug A at some active dose is combined with drug B at a dose that by itself has no effect. In our hybrid approach, we set out to test what would happen if we study the full dose response of drug A with and without the presence of no-effect or low-effect doses of drug B. As covered in the Results and Discussion below, this methodology ends up with significant advantages.

To begin with, we defined the dose response of hookworm *A*. *ceylanicum* infections in immunocompetent hamsters to each of these anti-nematode compounds. This parasite was chosen 1) because hookworms are the most important of the STNs, 2) because *A*. *ceylanicum* is a human pathogen and is increasingly recognized as major human hookworm parasite in Southeast Asia [52–54], 3) because of its close phylogenetic relationship to the other major hookworm parasites of humans (*Ancylostoma duodenale* and *Necator americanus* [55]), and 4) because it is a very good system for hookworm disease study [33].

**Fig 1:**
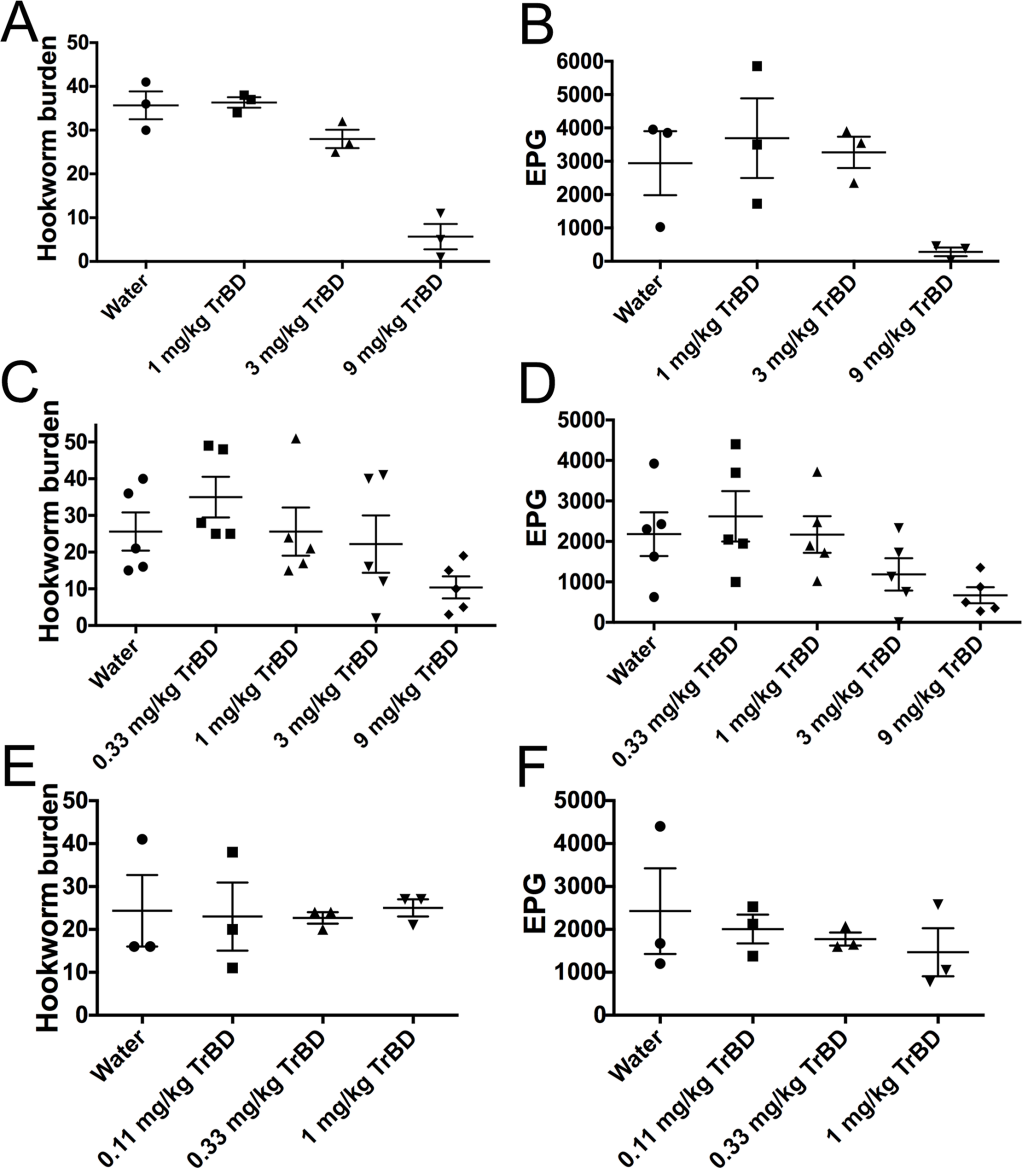
Dose response of tribendimidine against hookworm *A*. *ceylanicum* infections in hamsters. (A), (C), and (E): Effects of tribendimidine at indicated doses on intestinal hookworm burdens in hookworm-infected hamsters. For (A), (C), and (E) and in similar figures below, the hookworm burdens in each hamster are indicated with a separate symbol, long horizontal bars represent mean hookworm burdens per group; small bars indicate standard error. (B), (D), and (F): Effects of tribendimidine at indicated doses on parasite egg production (fecal egg counts) in hookworm-infected hamsters. For (B), (D), and (F) and in similar figures below, shown are the average eggs per gram of feces in each group on day 4 post-treatment. The fecal egg counts in each animal are indicated with a separate symbol. Long horizontal bars represent mean eggs per gram of feces (EPG) per group; small bars indicate standard error. Panel (A) & (B); (C) & (D) and (E) & (F) came from three independent *in vivo* experiments. TrBD = tribendimidine.

We performed dose response of *A*. *ceylanicum* infections in hamsters to tribendimidine (Fig 1). We chose 3X change between doses for these studies since it allows for getting a good range of doses with a fixed number of groups (*e*.*g*., vs. 2X change between doses) while still giving good discrimination of dose-responsiveness (*e*.*g*., vs. 10X change between doses). We chose as our upper dose (strong effect) 9 mg/kg tribendimidine based on previous literature [47] and went down factors of three to include 3 mg/kg and 1 mg/kg.

As shown in Fig 1A and 1B and S1 Table, a dose response to tribendimidine is seen. At 1 mg/kg tribendimidine relative to water control, the changes seen (actually 2% increase in hookworm burdens, P = 0.66 and 25.5% increase in fecal egg counts, P = 0.65) are not significant and within the normal variation given the group size. At 3 mg/kg tribendimidine, a reduction (21.6%) in hookworm burdens is seen that is not significant but approaches significance (P = 0.063). At 9 mg/kg tribendimidine, a large and significant reduction (84%; P = 0.008) in hookworm burdens is seen. These trends were largely confirmed in independent experiments that also included lower doses of the drug (Fig 1C, 1D, 1E and 1F; S1 Table). Based on these data, we concluded that 0.33 mg/kg and 1 mg/kg tribendimidine are the highest doses in this experiment that show no detectable impact on the parasites and are safe to use as “no effect doses” (as noted above, although 3 mg/kg tribendimidine data did not achieve statistical significance, this dose did appear to give a weak and reproducible impact on the parasites). These data also provide a good range (0.33–9 mg/kg) of tribendimidine doses that define a range of no effect to strong effect.

**Fig 2:**
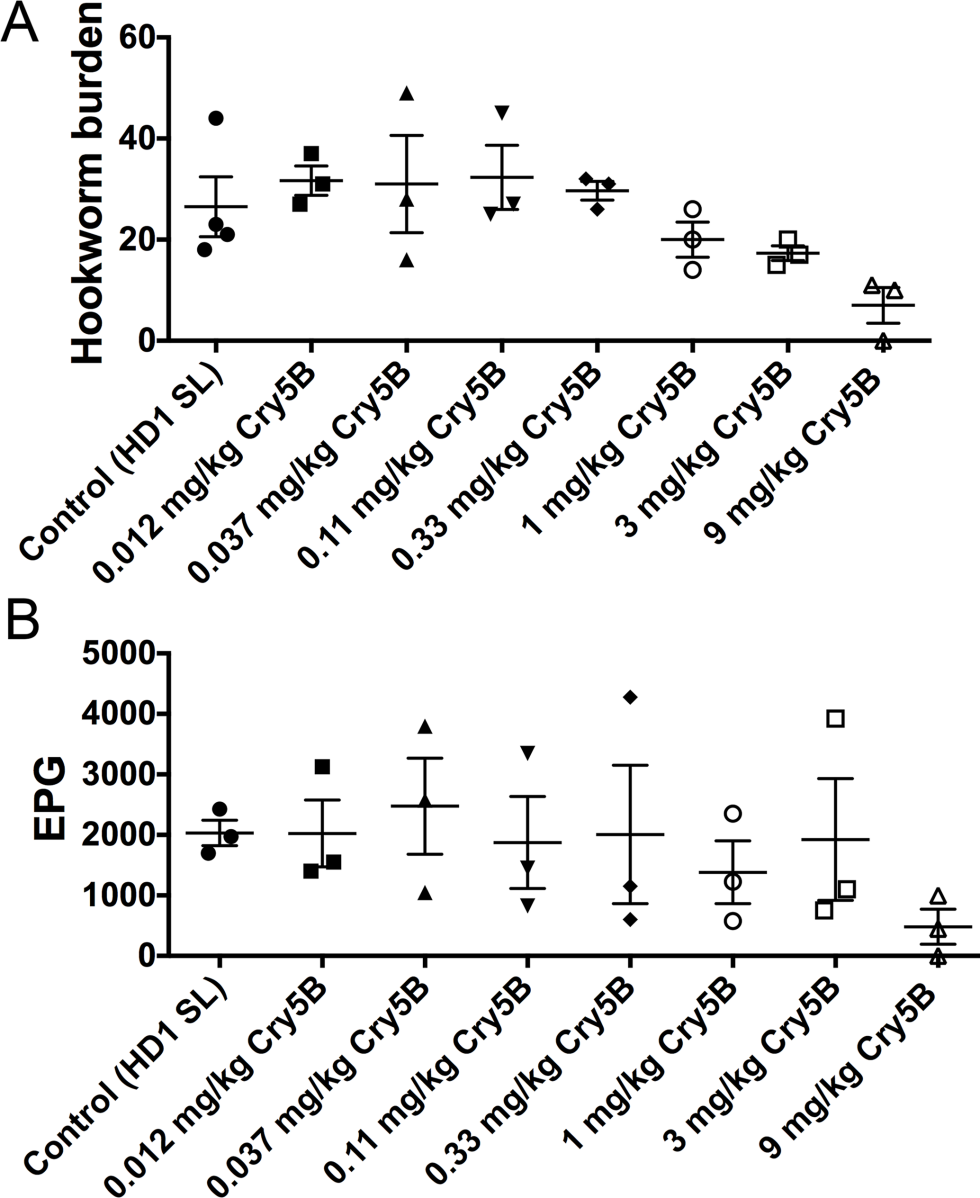
Dose response of Cry5B spore-crystal lysates (SCL) against hookworm *A*. *ceylanicum* infections in hamsters. Effects of Cry5B SCL at indicated doses on (A) intestinal hookworm burdens and (B) fecal egg counts in hookworm-infected hamsters.

**Fig 3:**
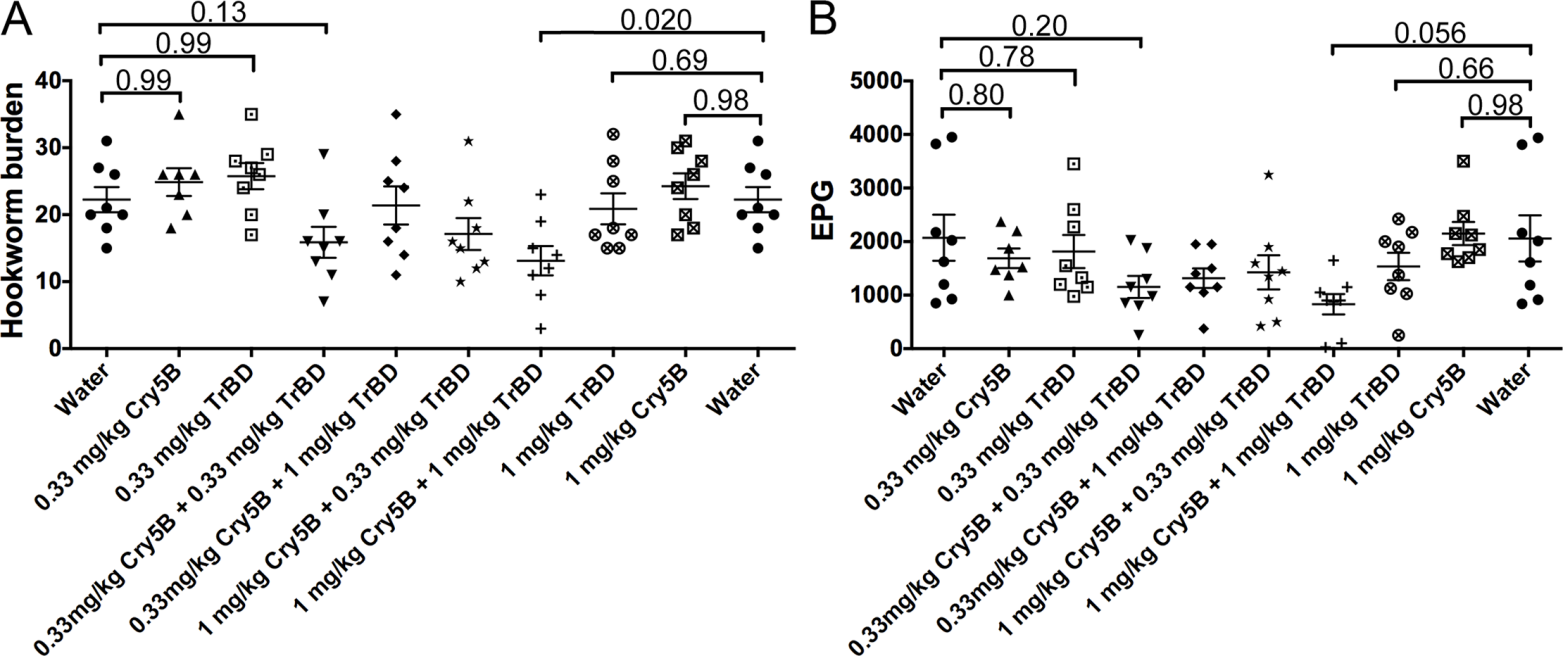
Cry5B and tribendimidine in combination are synergistic *in vivo* against hookworm infections in hamsters. Effects of individual treatment of Cry5B alone or tribendimidine alone and combination treatment of Cry5B plus tribendimidine at indicated doses on (A) intestinal hookworm burdens and (B) fecal egg counts in hookworm-infected hamsters. Brackets indicate statistical comparisons between groups, with p values shown. Data come from the combination of two independent *in vivo* experiments. TrBD = tribendimidine.

We similarly performed dose response of *A*. *ceylanicum* infections in hamsters to Cry5B (delivered as SCL from *Bacillus thuringiensis* [37]). No detectable impact on hookworm burdens or fecal egg counts was seen with Cry5B relative to SL control at 0.33 mg/kg Cry5B (12% increase in hookworm burdens, P = 0.98; 1.2% decrease in fecal egg counts, P = 0.81; Fig 2A and 2B; S1 Table). A small but not significant impact was seen at 1 mg/kg Cry5B (25% reduction in hookworm burdens, P = 0.46; 32% reduction in fecal egg counts, P = 0.45; Fig 2A and 2B; S1 Table). Previous experiments in the lab suggested that 1 mg/kg Cry5B delivered as SCLs does not have a significant impact, which was confirmed in subsequent experiments in this study (Figs 3 and 6). Although 3 mg/kg Cry5B did not have a statistically significant impact, it did seem to be part of a trend in increased efficacy between 1 mg/kg Cry5B and 9 mg/kg Cry5B, which did have a significant impact (74% reduction in hookworm burdens, P = 0.05; Fig 2A and 2B; S1 Table). Based on these data, we chose the 0.33 mg/kg and 1 mg/kg Cry5B doses as the highest doses that show no detectable impact on the parasites and 0.33 mg/kg up to 9 mg/kg as a range of Cry5B doses that define a range of no effect to strong effect.

A dose response of Cry5B (0.33, 1.0, 3.0 and 9.0 mg/kg) to hookworm infections in hamsters was then carried out without and with the two no-effect doses of tribendimidine (0.33 and 1.0 mg/kg), measuring hookworm burdens and fecal egg counts (S1A and S1B Fig; S2 Table). These combinations give Cry5B:tribendimidine ratios ranging from 1:3–27:1. We also carried out the reciprocal experiment in which a dose response of tribendimidine (0.33, 1.0, 3.0 and 9.0 mg/kg) to hookworm infections in hamsters was then carried out without and with the two no-effect doses of Cry5B (0.33 and 1.0 mg/kg), measuring hookworm burdens and fecal egg counts (S1C and S1D Fig; S2 Table).

Qualitatively, certain trends were visible. Most notably, whereas neither 0.33 mg/kg tribendimidine nor 0.33 mg/kg Cry5B gave any reduction in hookworm burden in either experiment relative to water control (in all cases the burdens were 2–27% higher than water control), the combination of 0.33 mg/kg tribendimidine plus 0.33 mg/kg Cry5B gave a 26%-31% reduction in hookworm burdens relative to water control (S2 Table). This (0.33 mg/kg + 0.33 mg/kg) combination showed better efficacy than 1 mg/kg of Cry5B alone (0%-8% reduction), 1 mg/kg tribendimidine alone (0%-18% reduction), or some other combinations, such as 0.33 mg Cry5B plus 1 mg/kg tribendimidine (0%-20% reduction) or 1 mg Cry5B plus 0.33 mg/kg tribendimidine (22%-24% reduction), all of which have more drug. Similarly, 1 mg/kg Cry5B alone or 1 mg/kg tribendimidine alone had no significant impact (0–18% reduction) relative to placebo (water) whereas the combination of the two at 1 mg/kg again showed a qualitatively higher impact (33%-49% reduction).

To increase the statistical power of our analyses, we combined the data from both experiments in which the same doses of each drug alone or in combination were given in both experiments (combining the data doubles the number of animals in each condition, which increases power). As shown (Fig 3A and 3B; Table 1), whereas none of the individual treatments (0.33 mg/kg of either Cry5B or tribendimidine or 1.0 mg/kg of either Cry5B or tribendimidine) were significantly different than placebo (P≥0.69 for hookworm burden 0%-6% reduction; P≥0.66 for fecal egg counts, 0%-26% reduction), the combination of Cry5B 1 mg/kg plus tribendimidine 1 mg/kg was significantly different from placebo control (for hookworm burdens 41% reduction, P = 0.020; for fecal egg counts 60% reduction, P = 0.056). These analyses show the two compounds are statistically synergistic. The other combination at 1:1 ratio (0.33 mg/kg Cry5B plus 0.33 mg/kg tribendimidine) approached statistical significance: P = 0.13 and 0.20 for hookworm burden (29% reduction) and fecal egg counts (44% reduction) respectively. In addition, this combination showed better efficacy in terms of both hookworm burden and fecal egg counts compared to either individual drug alone at a 3X higher dose of 1 mg/kg (0%-6% reduction in hookworm burdens; 0%-26% reduction in fecal egg counts). Both of the other combination ratios present in this combined analyses (1:3 and 3:1 tribendimidine:Cry5B) were also inferior in terms of their impact (4%-23% reduction in hookworm burdens and 31%-37% reduction in fecal egg counts) and P values relative to either equal ratio combination of (0.33 mg/kg + 0.33 mg/kg) or (1 mg/kg + 1 mg/kg) (Fig 3A and 3B; Table 1; see Discussion).

### 1:1 ratio of Cry5B:tribendimidine drives cures from incomplete to complete

**Fig 4:**
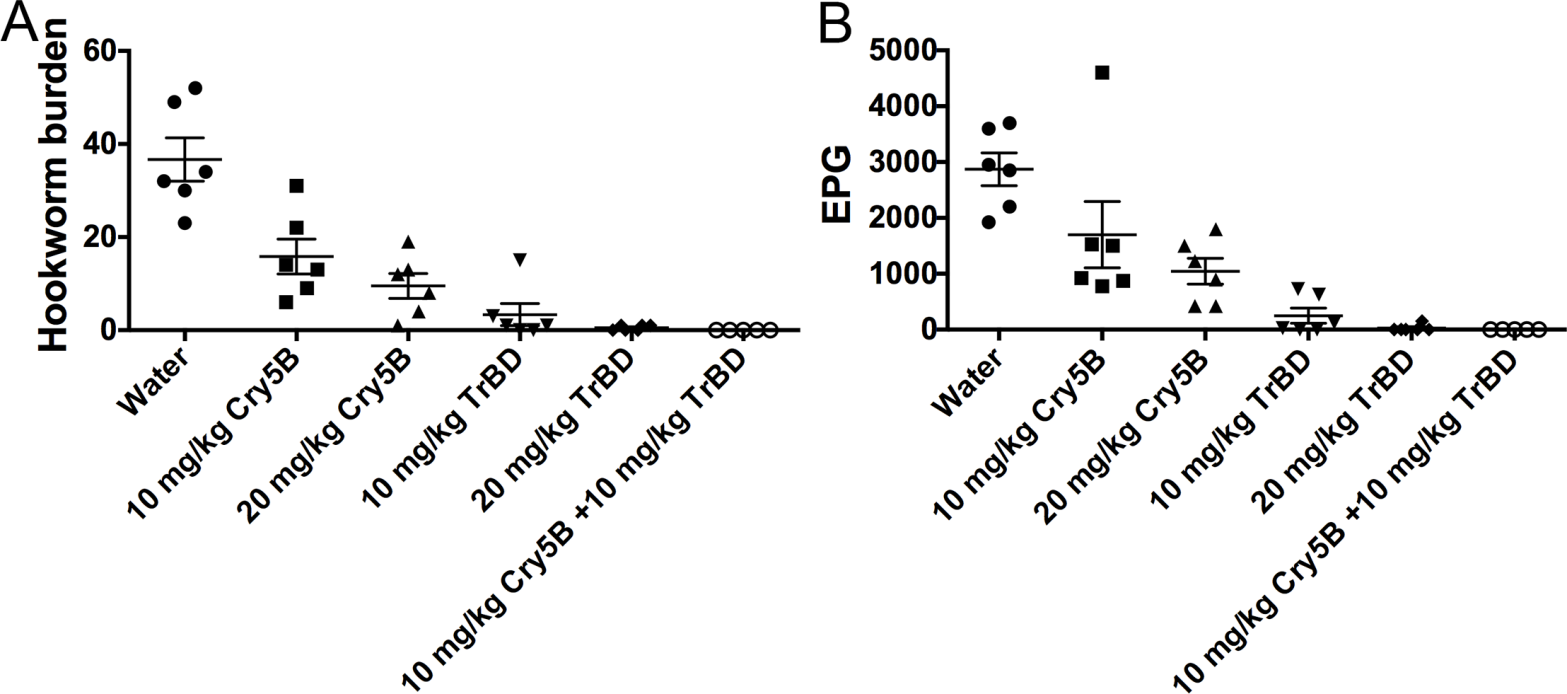
The 1:1 Cry5B:tribendimidine combination at a higher dose eliminates hookworm infections in hamsters. Effects of treatment of higher dose Cry5B alone or higher dose tribendimidine alone or higher dose Cry5B plus higher dose tribendimidine in combination on (A) intestinal hookworm burdens and (B) fecal egg counts in hookworm-infected hamsters. None of the individual drugs eradicated the hookworm infections in hamsters, but the combination of both drugs did. TrBD = tribendimidine.

These data demonstrate that a 1:1 mass ratio of the two drugs is highly synergistic. To see if we could apply this information in a meaningful way, we tested whether the two compounds can be combined at this ratio to drive an incomplete cure, which happens quite often in mass drug administration of anti-nematode drugs, to complete cures. As shown in Fig 4A and 4B and S1 Table, either 10 mg/kg of Cry5B alone or 10 m/kg of tribendimidine alone gave incomplete reductions in hookworm burdens (57% and 91%, respectively) and fecal egg counts (41% and 91%, respectively). However, the combination of 10 mg/kg of Cry5B plus 10 m/kg of tribendimidine resulted in a complete cure (100% reduction) of the parasites based on hookworm burdens and fecal egg counts (Fig 4A and 4B; S1 Table). The combination of 10 mg/kg Cry5B plus 10 mg/kg tribendimidine was even superior to 20 mg/kg of either compound alone (64%-99% reduction in hookworm burdens and fecal egg counts).

### Cry5B and pyrantel are synergistic *in vivo*

**Fig 5:**
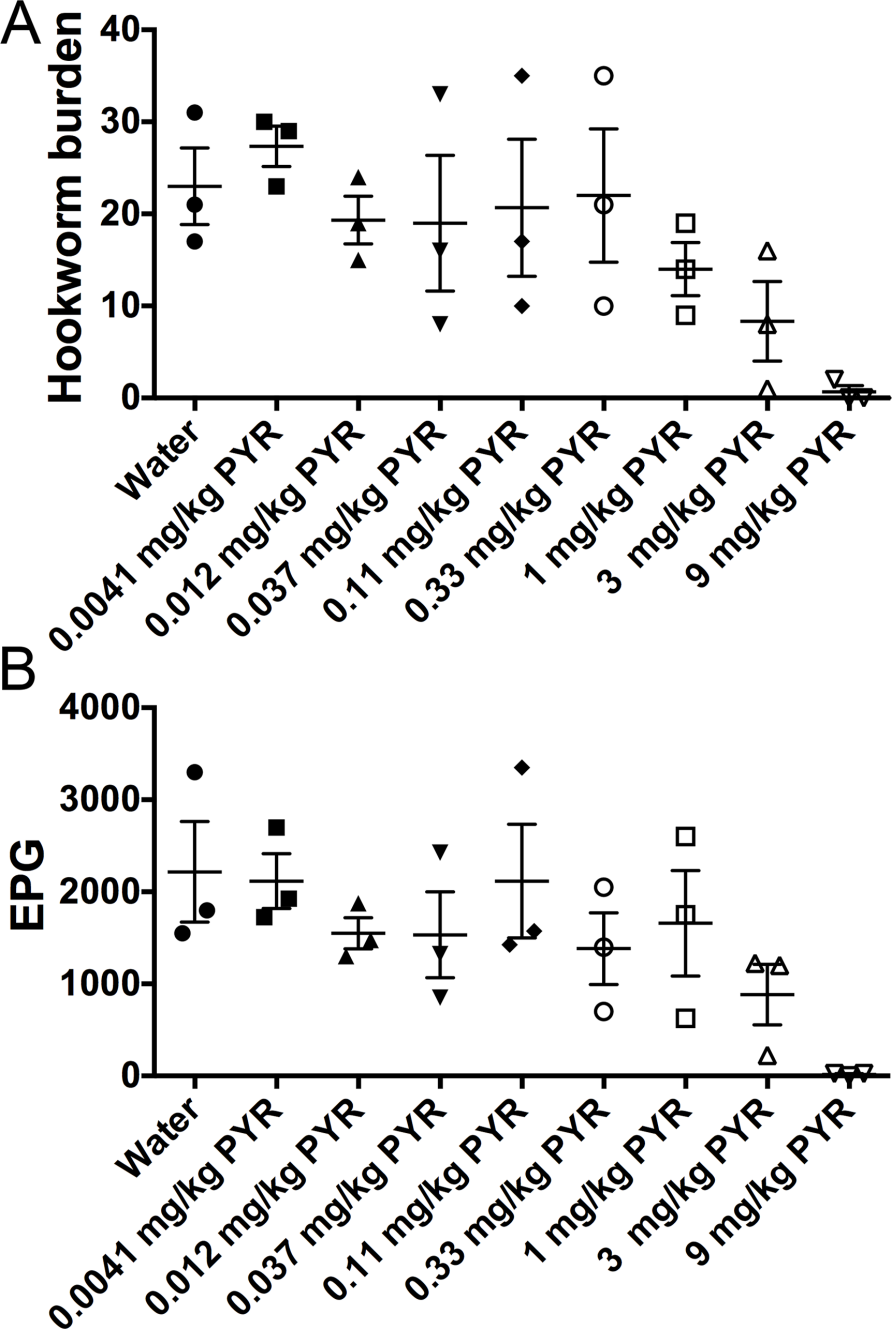
Dose response of pyrantel against hookworm *A*. *ceylanicum* infections in hamsters. Effects of pyrantel at indicated doses on (A) intestinal hookworm burdens and (B) fecal egg counts in hookworm-infected hamsters. PYR = pyrantel.

Although tribendimidine has good potential as a single dose anti-nematode drug for treatment of hookworms and *Ascaris* [15–17] it is nonetheless not yet a WHO approved drug. The mechanistically related nAChR agonist, pyrantel, on the other hand, is WHO approved. Although safe, pyrantel is not widely used because it has lower efficacy than BZs and is less conveniently dosed based on weight, versus fixed dosage like BZs [56]. To see if Cry5B synergizes with pyrantel *in vivo*, we performed a similar study to that used for tribendimidine, beginning with an *in vivo* pyrantel dose-response curve. We chose as our upper dose (strong effect) 9 mg/kg pyrantel based on previous literature [57] and then reduced subsequent doses by factors of three. Relative to water (placebo) control, 0.33 mg/kg pyrantel (labeled PYR in the figure) showed no significant impact on hookworm burdens (5% reduction, P = 0.87) whereas 1 mg/kg pyrantel showed a trend toward lower burdens that was not statistically significant (39% reduction, P = 0.22; Fig 5A; S1 Table). Looking at fecal egg counts, 0.33 mg/kg pyrantel showed a slight impact (38% reduction) that was not statistically significant (P = 0.47) from water control and was not different than other low doses like 0.037 mg/kg (31% reduction, P = 0.51; Fig 5B; S1 Table). Based on these data, we chose 0.33 mg/kg pyrantel as our no/low effect dose of this drug.

**Fig 6:**
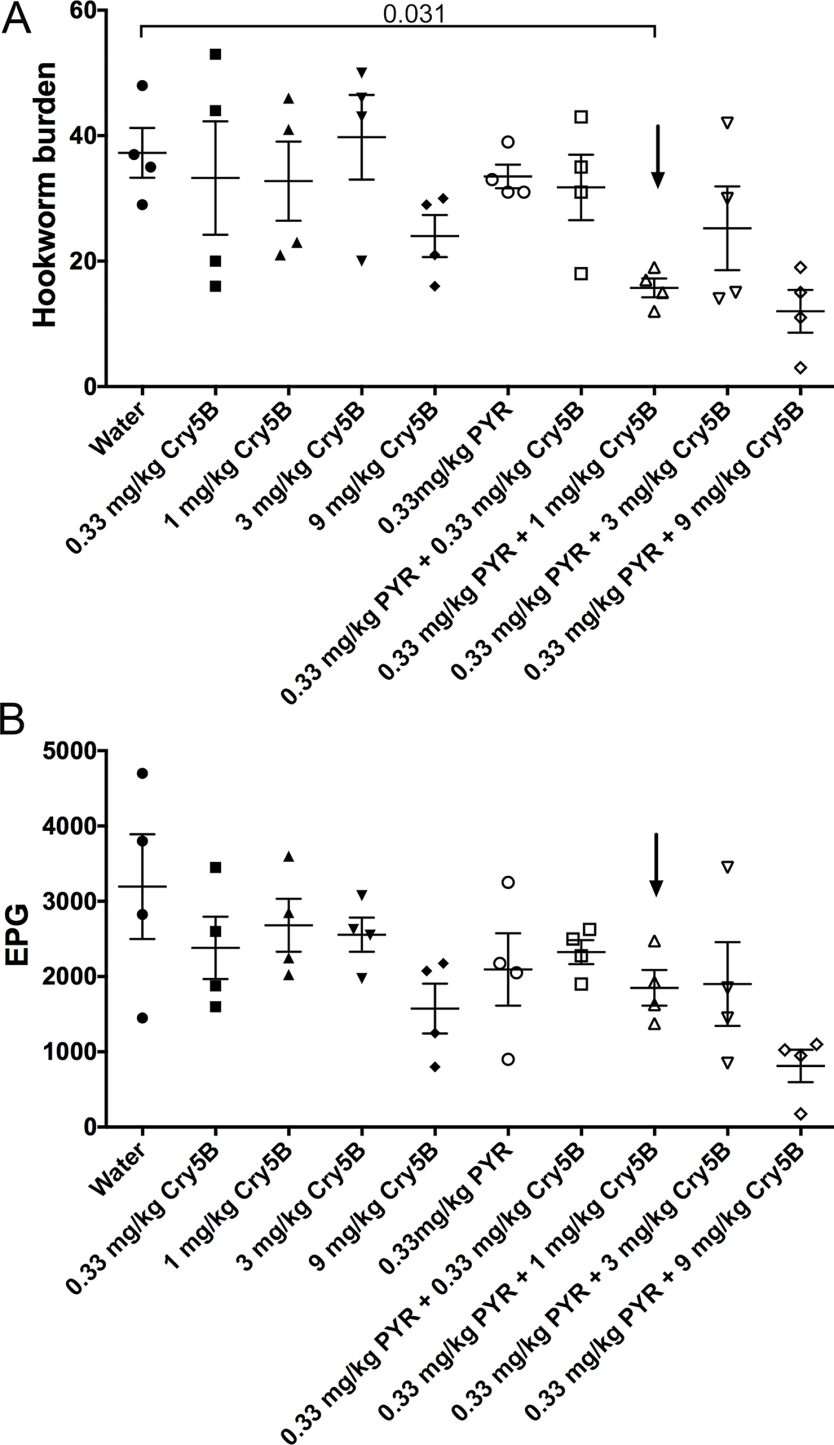
Cry5B and pyrantel in combination are synergistic *in vivo* against hookworm infections in hamsters. Effects of individual treatment of Cry5B alone or pyrantel alone and combination treatment of Cry5B plus pyrantel at indicated doses on (A) intestinal hookworm burdens and (B) fecal egg counts in hookworm-infected hamsters. Black arrow indicates the effect of the combination of Cry5B and pyrantel at ratio of 3:1 (1 mg/kg Cry5B + 0.33 mg/kg pyrantel), showing greater than expected impact based on individual doses or next highest combination dose. PYR = pyrantel.

A dose response of Cry5B (0.33, 1.0, 3.0 and 9.0 mg/kg) to hookworm infections in hamsters was then carried out without and with the chosen no/low effect dose of pyrantel (0.33 mg/kg), measuring hookworm burdens and fecal egg counts (Fig 6A and 6B; Table 2). Analyses of these data revealed a striking result. Combination of Cry5B and pyrantel at a 3:1 mass ratio (1 mg/kg Cry5B plus 0.33 mg/kg pyrantel) resulted in expulsion of parasites (58% reduction) that was beyond what was expected based on the effects of individual doses and statistically different from control (P = 0.031), even when each compound individually had no impact on hookworm burdens (10–12% reduction, P = 0.74–0.78; Fig 6A; Table 2). Indeed, 1 mg/kg Cry5B plus 0.33 mg/kg pyrantel was superior even to the next highest dose of 3 mg/kg Cry5B plus 0.33 mg/kg pyrantel (32% reduction, P = 0.35).

### 3:1 ratio of Cry5B:pyrantel drives cures from incomplete to complete

**Fig 7:**
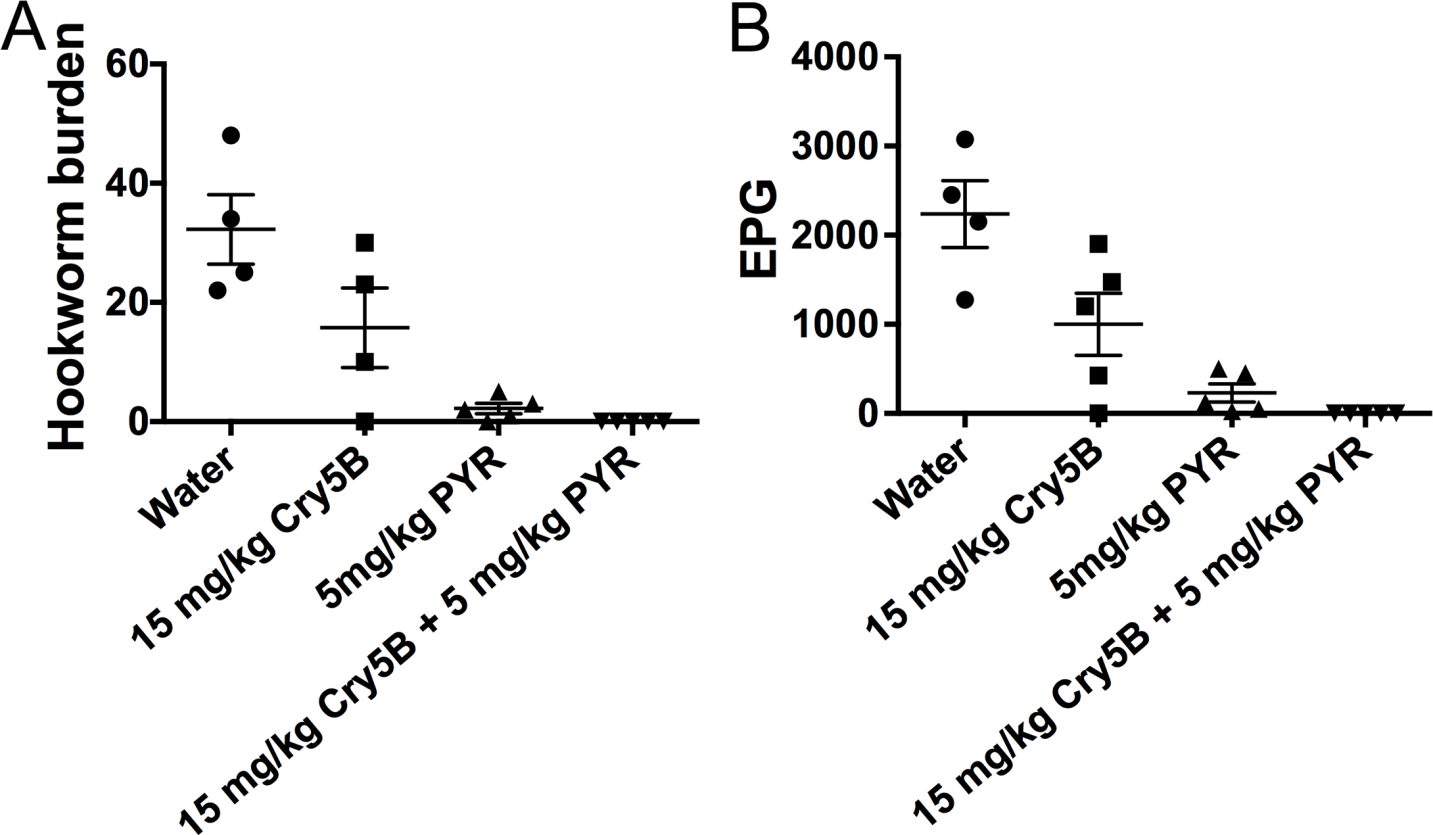
The 3:1 Cry5B:pyrantel combination at a higher dose eliminates hookworm infections in hamsters. Effects of treatment of higher dose Cry5B alone or higher dose pyrantel alone or higher dose Cry5B plus higher dose pyrantel in combination on (A) intestinal hookworm burdens and (B) fecal egg counts in hookworm-infected hamsters. None of the drugs individually eradicated the hookworm infections in hamsters but the combination of both drugs did. PYR = pyrantel.

These data demonstrate that a 3:1 mass ratio Cry5B:pyrantel is synergistic. As above, to see if we could apply this information in a meaningful way, we tested whether the two compounds could be combined at this ratio to drive incomplete cures to complete cures. As shown in Fig 7A and 7B and S1 Table, 15 mg/kg of Cry5B and 5 m/kg of pyrantel individually gave incomplete reductions in hookworm burdens (51% and 93%, respectively) and fecal egg counts (55% and 90%). However, the combination of the two resulted in a complete elimination of the parasites based on hookworm burdens and fecal egg counts (100% reductions; Fig 7A and 7B; S1 Table).

### A parasite population resistant to pyrantel is hypersusceptible to Cry5B relative to a non-resistant parasite population

In addition to synergy, another extremely useful property of drug combinations is hypersusceptibility or collateral sensitivity [58]. Hypersusceptibility or collateral sensitivity refers to the phenomenon whereby development of resistance by a microbe or cancer cell to one drug (drug A) results in increased sensitivity of the microbe or cancer to another drug (drug B) relative to the microbe (or cancer cell) not resistant to drug A. This relationship appears to be predictive of recalcitrance to resistance for both viruses and bacteria and is highly desirable [21,59–61]. We had previously demonstrated that *C*. *elegans* resistant to nAChR agonists are hypersusceptible to Cry5B and that *C*. *elegans* resistant to Cry5B are hypersusceptible to nAChR agonists [32].

**Fig 8:**
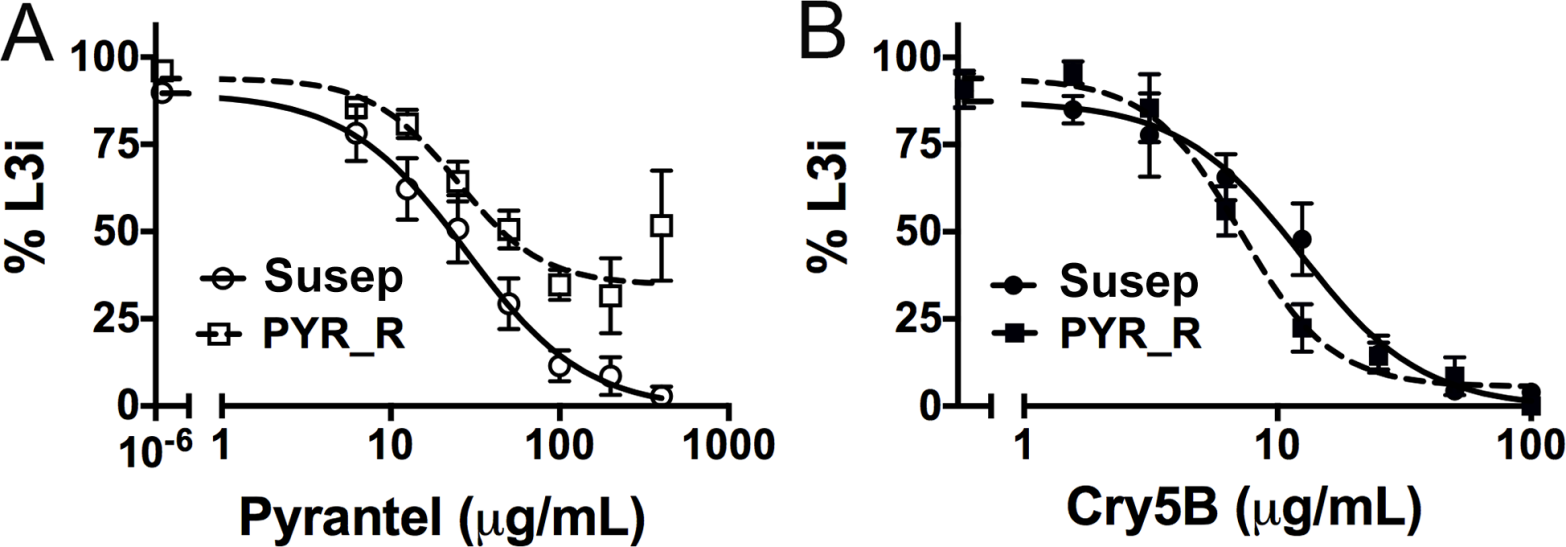
Pyrantel-resistant cyathostomins are hypersusceptible to Cry5B. Dose-dependent developmental inhibition of a non-anthelmintic resistant population of cyathostomins (Barn 10; [62]) and of an anthelmintic-resistant population of cyathostomins (pyrantel-resistant Population S or PYR_R) to (A) pyrantel and (B) purified Cry5B. Each data point represents the % of larvae that matured to the infectious L3 stage (L3i). Error bars indicate standard error from three independent replicates. Susep = Barn 10, pyrantel susceptible; PYR_R = pyrantel-resistant population.

To confirm whether the same relationship exists with parasitic nematodes, we compared the susceptibility of two populations of cyathostomins, small strongyle parasites of horse, one strain of which (known as Population S) has increased resistance to the nAChR agonist pyrantel, as well to BZs [36]. Cyathostomins are equine intestinal parasites related to human hookworms—both are clade V nematodes in the suborder Strongylida. Since live adult cyathostomin parasites are difficult to come by, we tested the ability of Cry5B to inhibit development of cyathostomin eggs (isolated from feces) to the infectious third larval stage. In this assay, we confirmed that, based on inhibitory concentration 50% (IC_50_) values, Population S is indeed resistant to pyrantel relative to the non-resistant population (Fig 8A; Table 3; P<0.001). Moreover, the pyrantel-resistant population, Population S, is hypersusceptible or hypersensitive to Cry5B based on IC_50_ values relative to the non-resistant population (Fig 8B; Table 3; P = 0.033).

## Discussion

Here we demonstrate that Cry5B forms potent and unprecedented anti-nematode combination therapy with the nAChR agonists tribendimidine and pyrantel. This finding is based on three findings. First, Cry5B synergizes with both drugs *in vivo*—a subtherapeutic dose of Cry5B combined with a subtherapeutic dose of tribendimidine or pyrantel results in significant reductions in hookworm burdens. This synergy appears at some, but not all, ratios of the compounds. Second, proper ratio mixing of Cry5B with either tribendimidine or pyrantel can drive incomplete cures to complete cures and can be superior to either drug alone at double the dose (tested with Cry5B and tribendimidine). Third, a parasite population resistant to nAChR agonists are hypersusceptible to Cry5B relative to a non-resistant population based on IC_50_ values. There are several caveats to these data. First, the two populations are not isogenic. Second, cyathostomin populations are a complex mixture of species. Third, the nAChR-resistant cyathostomin parasites tested (Populations S) are also resistant to BZs. BZ resistance could also contribute to the Cry5B hypersusceptible phenotype, although we noted that a *C*. *elegans* mutant resistant to BZs is not hypersusceptible to Cry5B [32]. Taken together, our cyathostomin data are consistent with our *C*. *elegans* data showing the hypersusceptibility of nAChR-resistant nematodes to Cry5B [32] and indicate that additional testing of nAChR-resistant parasite isolates is warranted to further confirm these results.

Based on our data, a 1:1 mass ratio of Cry5B:tribendimidine and 3:1 mass ratio of Cry5B:pyrantel shows more synergy than other ratios tested. This point is dramatically made by the finding that, for example, 0.33 mg/kg Cry5B plus 0.33 mg/kg tribendimidine is superior to 0.33 mg/kg Cry5B plus 1 mg/kg tribendimidine, despite the fact that the latter is higher in dose. Consistent with this finding, we find that 10 mg/kg Cry5B plus 10 mg/kg tribendimidine not only drives incomplete cures to complete cures but is superior to 20 mg/kg of either drug alone. Similarly for Cry5B plus pyrantel, 1 mg/kg Cry5B plus 0.33 mg/kg pyrantel is more potent than 3 mg/kg Cry5B plus 0.33 mg/kg pyrantel, despite the fact the latter is higher in dose.

These findings underscore the importance of setting the right ratios of drugs for combination therapy, as has been previously noted [63,64]. As we do not know the mechanism by which Cry5B and nAChR agonists might synergize, we can only speculate as to why some dose ratios are synergistic but others, including ones with higher doses, are not. As noted in Tardi et al., for example, cells can have different cellular responses to drug B at low dose versus at high dose. Drug A’s effects might be enhanced by the low-dose cellular response to drug B but not by the high-dose cellular response to drug B. Our data suggest efforts underway to combine anti-nematode drugs clinically [16,23–27] would benefit from preliminary studies such as those carried out here to determine which ratio of drugs achieves the best efficacy. By varying the ratios tested, it is possible that better clinical effects against parasites could be achieved than those seen now.

As for any infectious diseases (*e*.*g*., HIV, malaria, TB, STNs), resistance to drugs is of paramount importance. The hypersusceptibility to Cry5B seen here (also known as collateral sensitivity) for parasites resistant to nAChR agonists has important implications for resistance. Studies done with antibiotic-resistant bacteria predict that hypersusceptibility (or collateral sensitivity) is a highly desirable trait and correlates with delayed evolution of resistance to a drug combination [58,65,66]. Thus, we predict that Cry5B and nAChR agonists such as tribendimidine and pyrantel would have longer efficacy and delayed resistance.

Our synergy findings also have implications for the development of resistance. Synergy can either have a positive effect on delaying evolution of drug resistance or a negative effect (speeding up drug resistance) depending upon the effect that synergy has upon target microbe/parasite reproduction [67,68]. If the effects on reproduction of the target microbe/parasite are above a certain threshold, then synergy delays resistance (by limiting reproduction cycles). If, however, the effects on reproduction are below a threshold, then synergy speeds up resistance (since resistance to one of the drugs in the combination also removes the synergistic effect). In the case of Cry5B plus tribendimidine or pyrantel, our high dose combination data suggest that the synergy might have a positive effect on delaying resistance since hookworms are obligate parasites—complete elimination from the host blocks the reproductive cycle. Our high-dose results with Cry5B and nAChR agonists are consistent with recent modeling work that predicts the benefits of combining two anthelmintics at high efficacy doses with regards to delaying resistance [69,70].

In summary, we find that the combination of Cry5B with nAChR agonists, either tribendimidine or pyrantel, has powerful characteristics predicted to have excellent clinical efficacy and delayed resistance. Given that neither nAChR agonists nor Cry5B are widely used therapeutically against STNs, it would be ideal to deploy the combination as soon as possible to get the maximal benefit from the therapy before the parasites have time to develop resistance to either one. Furthermore, given the widespread use of nAChR agonists in veterinary medicine, Cry5B combinations could provide significant benefits for animal husbandry where anti-nematode drug resistance is rampant.

## Supporting information

S1 Fig Cry5B and tribendimidine (TrBD) combination studies *in vivo* against hookworm infections in hamsters. Hookworm burdens (A) and fecal egg counts (B) of infected hamsters treated with a range of Cry5B doses alone and in combination with two no-effect doses of TrBD. Hookworm burdens (C) and fecal egg counts (D) of infected hamsters treated with a range of TrBD doses alone and in combination with two no-effect doses of Cry5B. Arrows indicate the combinations of Cry5B and TrBD at ratio of 1:1 (0.33 mg/kg Cry5B + 0.33 mg/kg Cry5B and 1 mg/kg Cry5B + 1 mg/kg Cry5B), showing stronger-than-expected reductions in both measures of parasitism.
(DOCX)

S1 Table *In vivo* data associated with experimental results in Figs 1, 2, 4, 5 and 7. (DOCX)

S2 Table *In vivo* data associated with experimental results in S1 Fig. (DOCX)

S3 Table 95% confidence limits associated with experimental results in Tables 1, 2, S1 and S2. (DOCX)
