# Supplementary material for: In vivo and in vitro studies of Cry5B and nicotinic acetylcholine receptor agonist anthelmintics reveal a powerful and unique combination therapy against intestinal nematode parasites
Source: PLoS Negl Trop Dis. 2018 May 18;12(5):e0006506. doi: 10.1371/journal.pntd.0006506 (PMC5979042; doi:10.1371/journal.pntd.0006506)
Supplement: S1 Fig — Hookworm burdens (A) and fecal egg counts (B) of infected hamsters treated with a range of Cry5B doses alone and in combination with two no-effect doses of TrBD. Hookworm burdens (C) and fecal egg counts (D) of infected hamsters treated with a range of TrBD doses alone and in combination with two no-effect doses of Cry5B. Arrows indicate the combinations of Cry5B and TrBD at ratio of 1:1 (0.33 mg/kg Cry5B + 0.33 mg/kg Cry5B and 1 mg/kg Cry5B + 1 mg/kg Cry5B), showing stronger-than-expected reductions in both measures of parasitism. (DOCX) [file pntd.0006506.s001.docx]

**Supporting information**

Fig S1
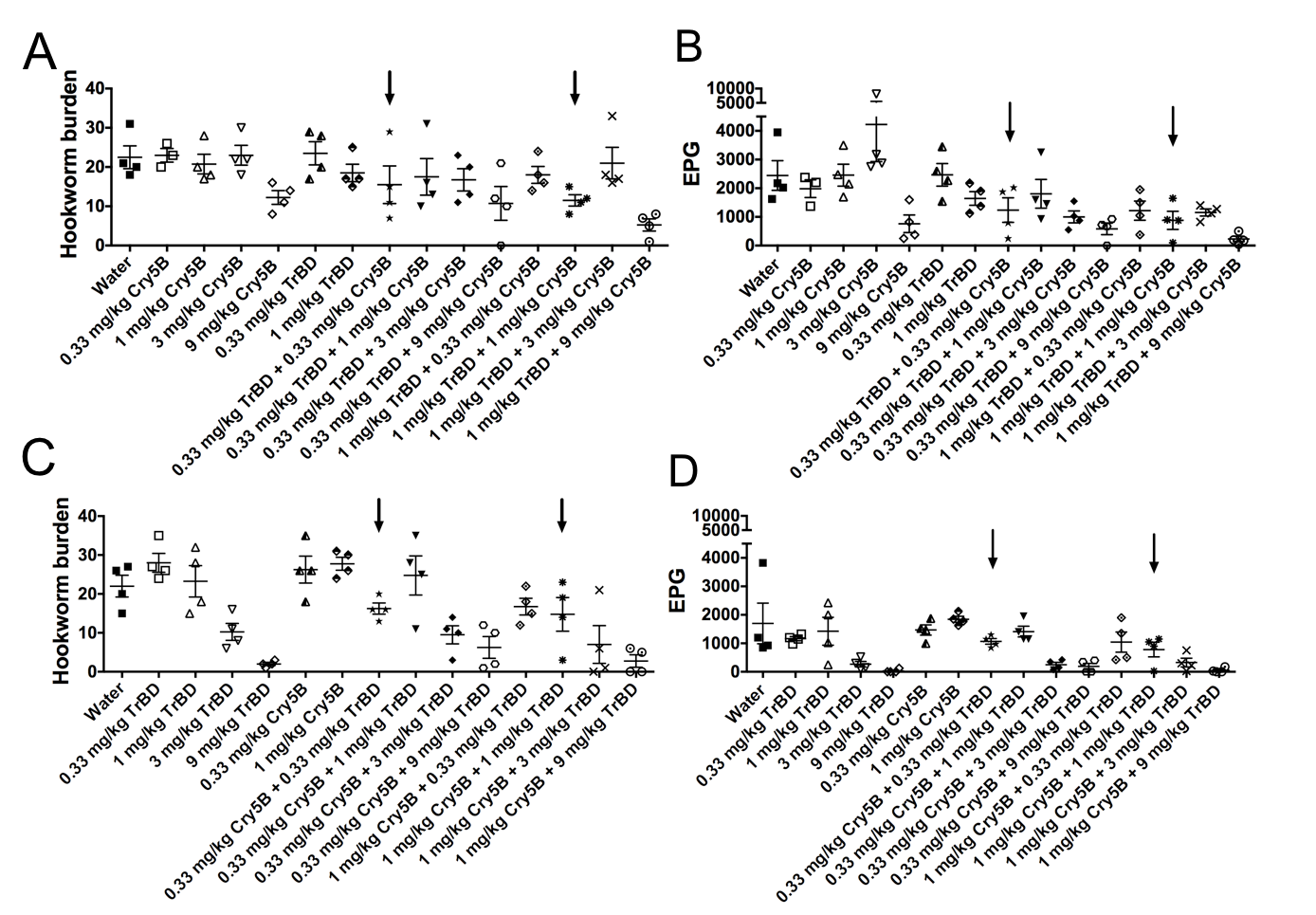


**Fig S1. Cry5B and tribendimidine (TrBD) combination studies *in vivo* against hookworm infections in hamsters.** Hookworm burdens (A) and fecal egg counts (B) of infected hamsters treated with a range of Cry5B doses alone and in combination with two no-effect doses of TrBD. Hookworm burdens (C) and fecal egg counts (D) of infected hamsters treated with a range of TrBD doses alone and in combination with two no-effect doses of Cry5B. Arrows indicate the combinations of Cry5B and TrBD at ratio of 1:1 (0.33 mg/kg Cry5B + 0.33 mg/kg Cry5B and 1 mg/kg Cry5B + 1 mg/kg Cry5B), showing stronger-than-expected reductions in both measures of parasitism.
