# Supplementary material for: In vivo and in vitro studies of Cry5B and nicotinic acetylcholine receptor agonist anthelmintics reveal a powerful and unique combination therapy against intestinal nematode parasites
Source: PLoS Negl Trop Dis. 2018 May 18;12(5):e0006506. doi: 10.1371/journal.pntd.0006506 (PMC5979042; doi:10.1371/journal.pntd.0006506)
Supplement: S1 Table — (DOCX) [file pntd.0006506.s002.docx]

**Table S1. *In vivo* data associated with experimental results in Figures 1, 2, 4, 5, and 7.**

| **Figure** | **Treatment** | **Hookworm burden (% reduction)** ^a^ | **P** ^b^ | **P** ^c^ | **Fecal egg counts**  **(% reduction)** ^d^ | **P** ^b^ | **P** ^c^ |
| --- | --- | --- | --- | --- | --- | --- | --- |
| 1A,B | Control (water) | 35.7 | na | na | 2942 | na | na |
| 1A,B | 1 mg/kg TrBD | 36.3 (-1.7) | 0.66 | 0.81 | 3692 (25.5) | 0.65 | 0.92 |
| 1A,B | 3 mg//kg TrBD | 28.0 (21.6) | 0.063 | 0.069 | 3267 (11.0) | 0.65 | 0.84 |
| 1A,B | 9 mg/kg TrBD | 5.7 (84.0) | 0.008 | <0.001 | 283 (90.4) | 0.037 | 0.056 |
| 1C,D | Control (water) | 25.6 | na | na | 2180 | na | na |
| 1C,D | 0.33 mg/kg TrBD | 35.0 (-36.7) | 0.99 | 0.98 | 2620 (-20.2) | 0.86 | 0.95 |
| 1C,D | 1 mg/kg TrBD | 25.6 (0) | 0.82 | 0.80 | 2170 (0.5) | 0.78 | 0.80 |
| 1C,D | 3 mg/kg TrBD | 22.2 (13.3) | 0.61 | 0.64 | 1185 (45.6) | 0.26 | 0.20 |
| 1C,D | 9 mg/kg TrBD | 10.4 (59.4) | 0.044 | 0.12 | 670 (69.3) | 0.042 | 0.051 |
| 1E,F | Control (water) | 24.3 | na | na | 2425 | na | na |
| 1E,F | 0.11 mg/kg TrBD | 23.0 (5.4) | 0.65 | 0.69 | 2008 (17.2) | 0.77 | 0.55 |
| 1E,F | 0.33 mg/kg TrBD | 22.7 (6.6) | 0.74 | 0.67 | 1775 (26.8) | 0.61 | 0.43 |
| 1E,F | 1 mg/kg TrBD | 25.0 (-2.9) | 0.86 | 0.78 | 1467 (39.5) | 0.49 | 0.30 |
| 2A,B | Control (SL) | 26.5 | na | na | 2033 | na | na |
| 2A,B | 0.012 mg/kg Cry5B | 31.7 (-19.6) | 0.99 | 0.99 | 2025 (0.4) | 0.88 | 0.87 |
| 2A,B | 0.037 mg/kg Cry5B | 31.0 (-17.0) | 0.91 | 0.98 | 2475 (-21.7) | 0.95 | 0.95 |
| 2A,B | 0.11 mg/kg Cry5B | 32.3 (-21.9) | 0.96 | 0.99 | 1875(7.8) | 0.78 | 0.83 |
| 2A,B | 0.33 mg/kg Cry5B | 29.7 (-12.1) | 0.98 | 0.97 | 2008 (1.2) | 0.81 | 0.87 |
| 2A,B | 1 mg/kg Cry5B | 20.0 (24.5) | 0.46 | 0.55 | 1383 (32.0) | 0.45 | 0.65 |
| 2A,B | 3 mg/kg Cry5B | 17.3 (34.7) | 0.18 | 0.38 | 1925 (5.3) | 0.77 | 0.85 |
| 2A,B | 9 mg/kg Cry5B | 7.0 (73.6) | 0.050 | 0.035 | 483 (76.2) | 0.037 | 0.27 |
| 4A,B | Control (water) | 36.7 | na | na | 2871 | na | na |
| 4A,B | 10 mg/kg Cry5B | 15.8 (56.9) | 0.007 | <0.001 | 1700 (40.8) | 0.068 | 0.014 |
| 4A,B | 20 mg/kg Cry5B | 9.5 (74.1) | <0.001 | <0.001 | 1046 (63.6) | <0.001 | <0.001 |
| 4A,B | 10 mg/kg TrBD | 3.3 (90.9) | 0.002 | <0.001 | 250 (91.3) | <0.001 | <0.001 |
| 4A,B | 20 mg/kg TrBD | 0.5 (98.6) | <0.001 | <0.001 | 25 (99.1) | <0.001 | <0.001 |
| 4A,B | 10 mg/kg Cry5B  + 10 mg/kg TrBD | 0 (100) | <0.001 | <0.001 | 0 (100) | <0.001 | <0.001 |
| 5A,B | Control (water) | 23.0 | na | na | 2217 | na | na |
| 5A,B | 0.0041 mg/kg PYR | 27.3 (-18.8) | 0.99 | 0.98 | 2117 (4.5) | 0.94 | 0.85 |
| 5A,B | 0.012 mg/kg PYR | 19.3 (15.9) | 0.68 | 0.72 | 1550 (30.1) | 0.44 | 0.45 |
| 5A,B | 0.037 mg/kg PYR | 19.0 (17.4) | 0.68 | 0.70 | 1533 (30.8) | 0.51 | 0.44 |
| 5A,B | 0.11 mg/kg PYR | 20.7 (10.1) | 0.80 | 0.79 | 2117 (4.5) | 0.83 | 0.85 |
| 5A,B | 0.33 mg/kg PYR | 22.0 (4.3) | 0.87 | 0.85 | 1383 (37.6) | 0.47 | 0.33 |
| 5A,B | 1 mg/kg PYR | 14.0 (39.1) | 0.22 | 0.38 | 1658 (25.2) | 0.68 | 0.54 |
| 5A,B | 3 mg/kg PYR | 8.3 (63.8) | 0.079 | 0.12 | 883 (60.2) | 0.054 | 0.09 |
| 5A,B | 9 mg/kg PYR | 0.7 (97.1) | 0.028 | 0.01 | 17 (99.2) | 0.035 | 0.005 |
| 7A,B | Control (water) | 32.3 | na | na | 2238 | na | na |
| 7A,B | 15 mg/kg Cry5B | 15.8 (51.2) | 0.20 | 0.017 | 1000 (55.3) | 0.048 | 0.007 |
| 7A,B | 5 mg/kg PYR | 2.2 (93.2) | 0.003 | <0.001 | 230 (89.7) | <0.001 | <0.001 |
| 7A,B | 15 mg/kg Cry5B  + 5 mg/kg PYR | 0 (100.0) | <0.001 | <0.001 | 0 (100) | <0.001 | <0.001 |

^a^ Average hookworm burdens (% reduction relative to water control)

^b^ P value relative to water control, nonparametric Konietschke and Pauly comparison. See Materials and Methods.

^c^ P value relative to water control, parametric Dunnett’s (ANOVA) comparison. See Materials and Methods.

^d^ Average fecal egg counts burdens (% reduction relative to water control)

na: not applicable

TrBD=Tribendimidine

PYR=Pyrantel
