# Supplementary material for: In vivo and in vitro studies of Cry5B and nicotinic acetylcholine receptor agonist anthelmintics reveal a powerful and unique combination therapy against intestinal nematode parasites
Source: PLoS Negl Trop Dis. 2018 May 18;12(5):e0006506. doi: 10.1371/journal.pntd.0006506 (PMC5979042; doi:10.1371/journal.pntd.0006506)
Supplement: S2 Table — (DOCX) [file pntd.0006506.s003.docx]

**Table S2. *In vivo* data associated with experimental results in Figure S1.**

| **Figure** | **Treatment** | **Hookworm burden (% reduction)** ^a^ | **P** ^b^ | **P** ^c^ | **Fecal egg counts**  **(% reduction)** ^d^ | **P** ^b^ | **P** ^c^ |
| --- | --- | --- | --- | --- | --- | --- | --- |
| S1 A,B | Control (water) | 22.5 | na | na | 2444 | na | na |
| S1 A,B | 0.33 mg/kg TrBD | 23.5 (-4.4) | 0.97 | 0.97 | 2469 (-0.0) | 0.98 | 0.94 |
| S1 A,B | 1 mg/kg TrBD | 18.5 (17.8) | 0.50 | 0.64 | 1644 (32.7) | 0.38 | 0.50 |
| S1 A,B | 0.33 mg/kg Cry5B | 23.0 (-2.2) | 0.99 | 0.95 | 1983 (18.9) | 0.85 | 0.76 |
| S1 A,B | 1 mg/kg Cry5B | 20.8 (7.6) | 0.81 | 0.84 | 2456 (-0.0) | 0.98 | 0.94 |
| S1 A,B | 3 mg/kg Cry5B | 23.0 (-2.2) | 0.98 | 0.95 | 4225 (-0.7) | 0.99 | 1.00 |
| S1 A,B | 9 mg/kg Cry5B | 12.3 (45.3) | 0.049 | 0.09 | 763 (68.8) | 0.065 | 0.061 |
| S1 A,B | 0.33 mg/kg TrBD  + 0.33 mg/kg Cry5B | 15.5 (31.1) | 0.44 | 0.31 | 1238 (49.4) | 0.30 | 0.23 |
| S1 A,B | 0.33 mg/kg TrBD  + 1 mg/kg Cry5B | 17.5 (22.2) | 0.53 | 0.52 | 1806 (26.1) | 0.57 | 0.62 |
| S1 A,B | 0.33 mg/kg TrBD  + 3 mg/kg Cry5B | 16.8 (25.3) | 0.49 | 0.44 | 1000 (59.1) | 0.048 | 0.13 |
| S1 A,B | 0.33 mg/kg TrBD  + 9 mg/kg Cry5B | 10.8 (52.0) | 0.24 | 0.04 | 581 (76.2) | 0.020 | 0.033 |
| S1 A,B | 1 mg/kg TrBD  + 0.33 mg/kg Cry5B | 18.0 (20.0) | 0.49 | 0.58 | 1219 (50.1) | 0.18 | 0.22 |
| S1 A,B | 1 mg/kg TrBD  + 1 mg/kg Cry5B | 11.5 (48.9) | 0.036 | 0.06 | 881 (64.0) | 0.11 | 0.089 |
| S1 A,B | 1 mg/kg TrBD  + 3 mg/kg Cry5B | 21.0 (6.7) | 0.79 | 0.86 | 1156 (52.7) | 0.042 | 0.19 |
| S1 A,B | 1 mg/kg TrBD  + 9 mg/kg Cry5B | 5.3 (76.4) | 0.020 | <0.001 | 231 (90.5) | 0.008 | 0.009 |
| S1 C,D | Control (water) | 22.0 | na | na | 1700 | na | na |
| S1 C,D | 0.33 mg/kg Cry5B | 26.3 (-19.3) | 0.99 | 1.00 | 1469 (13.6) | 0.96 | 0.77 |
| S1 C,D | 1 mg/kg Cry5B | 27.8 (-26.1) | 0.99 | 1.00 | 1844 (-0.08) | 0.99 | 0.98 |
| S1 C,D | 0.33 mg/kg TrBD | 28.0 (-27.3) | 0.99 | 1.00 | 1163 (31.6) | 0.84 | 0.40 |
| S1 C,D | 1 mg/kg TrBD | 23.3 (-5.7) | 0.95 | 0.97 | 1425 (16.2) | 0.88 | 0.72 |
| S1 C,D | 3 mg/kg TrBD | 10.2(53.4) | 0.070 | 0.041 | 269 (84.2) | 0.068 | 0.003 |
| S1 C,D | 9 mg/kg TrBD | 2.0 (90.9) | 0.011 | <0.001 | 31.3 (98.2) | 0.025 | <0.001 |
| S1 C,D | 0.33 mg/kg Cry5B  + 0.33 mg/kg TrBD | 16.3 (26.1) | 0.32 | 0.44 | 1069 (37.1) | 0.68 | 0.29 |
| S1 C,D | 0.33 mg/kg Cry5B  + 1 mg/kg TrBD | 24.8 (-5.7) | 0.96 | 0.99 | 1413 (16.9) | 0.95 | 0.71 |
| S1 C,D | 0.33 mg/kg Cry5B  + 3 mg/kg TrBD | 9.5 (56.8) | 0.038 | 0.027 | 250 (85.3) | 0.063 | 0.002 |
| S1 C,D | 0.33 mg/kg Cry5B  + 9 mg/kg TrBD | 6.3 (71.6) | 0.032 | 0.004 | 188 (88.9) | 0.063 | 0.001 |
| S1 C,D | 1 mg/kg Cry5B  + 0.33 mg/kg TrBD | 16.8 (23.8) | 0.37 | 0.49 | 1044 (38.6) | 0.77 | 0.27 |
| S1 C,D | 1 mg/kg Cry5B  + 1 mg/kg TrBD | 14.8 (32.9) | 0.39 | 0.28 | 781 (54.1) | 0.42 | 0.080 |
| S1 C,D | 1 mg/kg Cry5B  + 3 mg/kg TrBD | 7.0 (68.2) | 0.16 | 0.86 | 325 (80.9) | 0.074 | 0.004 |
| S1 C,D | 1 mg/kg Cry5B  + 9 mg/kg TrBD | 2.8 (87.5) | 0.074 | <0.001 | 50 (97.1) | 0.029 | <0.001 |

^a^ Average hookworm burdens (% reduction relative to water control)

^b^ P value relative to water control, nonparametric Konietschke and Pauly comparison. See Materials and Methods.

^c^ P value relative to water control, parametric Dunnett’s (ANOVA) comparison. See Materials and Methods.

^d^ Average fecal egg counts burdens (% reduction relative to water control)

na: not applicable

TrBD=Tribendimidine
