# Supplementary material for: In vivo and in vitro studies of Cry5B and nicotinic acetylcholine receptor agonist anthelmintics reveal a powerful and unique combination therapy against intestinal nematode parasites
Source: PLoS Negl Trop Dis. 2018 May 18;12(5):e0006506. doi: 10.1371/journal.pntd.0006506 (PMC5979042; doi:10.1371/journal.pntd.0006506)
Supplement: S3 Table — (DOCX) [file pntd.0006506.s004.docx]

**Table S3. 95% confidence limits associated with experimental results in Tables 1, 2, S1, and S2.**

| **Figure** | **Treatment** | **Hookworm burden (95% C.L.)** | **Fecal egg counts**  **(95% C.L.)** |
| --- | --- | --- | --- |
| 1A,B | Control (water) | 35.6 (22.0,49.4) | 2942 (-1184,7067) |
| 1A,B | 1 mg/kg TrBD | 36.3 (31.2,41.5) | 3962 (-1448,8832) |
| 1A,B | 3 mg//kg TrBD | 28.0 (19.0,37.0) | 3267 (1247,5286) |
| 1A,B | 9 mg/kg TrBD | 5.7 (-6.8,18.2) | 283 (-280,847) |
| 1C,D | Control (water) | 25.6 (11.2,40.0) | 2180 (679,3681) |
| 1C,D | 0.33 mg/kg TrBD | 35.0 (19.6,50.4) | 2620 (894,4346) |
| 1C,D | 1 mg/kg TrBD | 25.6 (7.4,43.8) | 2170 (914,3426) |
| 1C,D | 3 mg/kg TrBD | 22.2 (0.5,43.9) | 1185 (76,2294) |
| 1C,D | 9 mg/kg TrBD | 10.4 (2.1,18.7) | 670 (118,1222) |
| 1E,F | Control (water) | 24.3 (-11.5,60.2) | 2425 (-1865,6715) |
| 1E,F | 0.11 mg/kg TrBD | 23.0 (-11.2,57.2) | 2008 (558,3459) |
| 1E,F | 0.33 mg/kg TrBD | 22.7 (16.9,28.4) | 1775 (1127,2423) |
| 1E,F | 1 mg/kg TrBD | 25.0 (16.4,33.6) | 1467 (-942,3875) |
| 2A,B | Control (SL) | 26.5 (7.7,45.4) | 2033 (1124,2943) |
| 2A,B | 0.012 mg/kg Cry5B | 31.7 (19.2,44.2) | 2025 (-349,4399) |
| 2A,B | 0.037 mg/kg Cry5B | 31.0 (-10.5,72.5) | 2475 (-948,5897) |
| 2A,B | 0.11 mg/kg Cry5B | 32.3 (5.0,59.7) | 1875(-1392,5142) |
| 2A,B | 0.33 mg/kg Cry5B | 29.7 (21.7,37.7) | 2008 (-2916,6932) |
| 2A,B | 1 mg/kg Cry5B | 20.0 (5.1,34.9) | 1383 (-848,3614) |
| 2A,B | 3 mg/kg Cry5B | 17.3 (11.1,23.6) | 1925 (-2400,6250) |
| 2A,B | 9 mg/kg Cry5B | 7.0 (-8.1,22.1) | 483 (-761,1727) |
| 3A,B | Control (water) | 22.3 (17.8,26.7) | 2072 (1054,3090) |
| 3A,B | 0.33 mg/kg Cry5B | 24.9 (19.8,29.9) | 1689 (1241,2138) |
| 3A,B | 1 mg/kg Cry5B | 24.3 (19.7,28.8) | 2150 (1637,2663) |
| 3A,B | 0.33 mg/kg TrBD | 25.8 (21.1,30.4) | 1816 (1086,2546) |
| 3A,B | 1 mg/kg TrBD | 20.9 (15.4,26.3) | 1534 (930,2139) |
| 3A,B | 0.33 mg/kg Cry5B  + 0.33 mg/kg TrBD | 15.9 (10.4,21.4) | 1153 (667,1639) |
| 3A,B | 0.33 mg/kg Cry5B  + 1 mg/kg TrBD | 21.4 (14.7,28.1) | 1316 (885,1746) |
| 3A,B | 1 mg/kg Cry5B  + 0.33 mg/kg TrBD | 17.1 (11.5,22.8) | 1425 (671,2179) |
| 3A,B | 1 mg/kg Cry5B  + 1 mg/kg TrBD | 13.1 (7.9,18.3) | 831 (383,1280) |
| 4A,B | Control (water) | 36.7 (24.7,48.6) | 2871 (2118,3623) |
| 4A,B | 10 mg/kg Cry5B | 15.8 (6.2,25.5) | 1700 (171,3229) |
| 4A,B | 20 mg/kg Cry5B | 9.5 (2.6,16.4) | 1046 (452,1639) |
| 4A,B | 10 mg/kg TrBD | 3.3 (-2.8,9.4) | 250 (-100,600) |
| 4A,B | 20 mg/kg TrBD | 0.5 (-0.07,1.1) | 25 (-39,89) |
| 4A,B | 10 mg/kg Cry5B  + 10 mg/kg TrBD | 0 (0,0) | 0 (0,0) |
| 5A,B | Control (water) | 23.0 (5.1,40.9) | 2217 (-135,4568) |
| 5A,B | 0.0041 mg/kg PYR | 27.3 (17.9,36.7) | 2117 (837,3396) |
| 5A,B | 0.012 mg/kg PYR | 19.3 (8.1,30.5) | 1550 (818,2282) |
| 5A,B | 0.037 mg/kg PYR | 19.0 (-12.7,50.7) | 1533 (-474,3540) |
| 5A,B | 0.11 mg/kg PYR | 20.7 (-11.4,52.7) | 2117 (-543,4777) |
| 5A,B | 0.33 mg/kg PYR | 22.0 (-9.1,53.1) | 1383 (-294,3061) |
| 5A,B | 1 mg/kg PYR | 14.0 (1.6,26.4) | 1658 (-803,4119) |
| 5A,B | 3 mg/kg PYR | 8.3 (-10.3,27.0) | 883 (-533,2300) |
| 5A,B | 9 mg/kg PYR | 0.7 (-2.2,3.5) | 17 (-19,53) |
| 6A,B | Control (water) | 37.3 (24.6,49.9) | 3194 (979,5409) |
| 6A,B | 0.33 mg/kg PYR | 33.5 (27.5,39.5) | 2094 (565,3623) |
| 6A,B | 0.33 mg/kg Cry5B | 33.3 (4.5,62.0) | 2381 (1064,3699) |
| 6A,B | 1 mg/kg Cry5B | 32.8 (12.7,52.8) | 2681 (1560,3802) |
| 6A,B | 3 mg/kg Cry5B | 39.8 (18.3,61.2) | 2556 (1838,3275) |
| 6A,B | 9 mg/kg Cry5B | 24.0 (13.4,34.6) | 1575 (521,2629) |
| 6A,B | 0.33 mg/kg PYR  + 0.33 mg/kg Cry5B | 31.8 (15.1,48.4) | 2325 (1819,2831) |
| 6A,B | 0.33 mg/kg PYR  + 1 mg/kg Cry5B | 15.8 (11.0,20.5) | 1850 (1097,2603) |
| 6A,B | 0.33 mg/kg PYR  + 3 mg/kg Cry5B | 25.3 (4.0,46.5) | 1900 (130,3670) |
| 6A,B | 0.33 mg/kg PYR  + 9 mg/kg Cry5B | 12.0 (1.1,22.9) | 813 (129,1496) |
| 7C,D | Control (water) | 32.3 (13.7,50.8) | 2238 (1047,3428) |
| 7C,D | 15 mg/kg Cry5B | 15.8 (-5.5,37.0) | 1000 (37.0,1963) |
| 7C,D | 5 mg/kg PYR | 2.2 (-0.2,4.6) | 230 (-52,512) |
| 7C,D | 15 mg/kg Cry5B  + 5 mg/kg PYR | 0 (0,0) | 0 (0,0) |
| S1A,B | Control (water) | 22.5 (13.3,31.7) | 2444 (804,4084) |
| S1A,B | 0.33 mg/kg TrBD | 23.5 (14.1,32.9) | 2469 1215,3722) |
| S1A,B | 1 mg/kg TrBD | 18.5 (11.4,25.6) | 1644 (881,2406) |
| S1A,B | 0.33 mg/kg Cry5B | 23.0 (15.6,30.5) | 1983 (657,3310) |
| S1A,B | 1 mg/kg Cry5B | 20.8 (12.8,28.7) | 2456 (1239,3673) |
| S1A,B | 3 mg/kg Cry5B | 23.0 (15.0,31.0) | 4225 (105,8345) |
| S1A,B | 9 mg/kg Cry5B | 12.3 (6.7,17.8) | 763 (-209,1734) |
| S1A,B | 0.33 mg/kg TrBD  + 0.33 mg/kg Cry5B | 15.5 (0.3,30.7) | 1238 (-123,2598) |
| S1A,B | 0.33 mg/kg TrBD  + 1 mg/kg Cry5B | 17.5 (2.7,32.3) | 1806 (207,3406) |
| S1A,B | 0.33 mg/kg TrBD  + 3 mg/kg Cry5B | 16.8 (7.7, 25.8) | 1000 (337,1663) |
| S1A,B | 0.33 mg/kg TrBD  + 9 mg/kg Cry5B | 10.8 (-3.0,24.5) | 581 (-59,1221) |
| S1A,B | 1 mg/kg TrBD  + 0.33 mg/kg Cry5B | 18.0 (11.1,24.9) | 1219 (150,2288) |
| S1A,B | 1 mg/kg TrBD  + 1 mg/kg Cry5B | 11.5 (6.9,16.1) | 881 (-126,1888) |
| S1A,B | 1 mg/kg TrBD  + 3 mg/kg Cry5B | 21.0 (8.2,33.8) | 1156 (762,1551) |
| S1A,B | 1 mg/kg TrBD  + 9 mg/kg Cry5B | 5.3 (0.3,10.2) | 231 (-72,535) |
| S1C,D | Control (water) | 22.0 (13.1,30.9) | 1700 (-567,3967) |
| S1C,D | 0.33 mg/kg Cry5B | 26.3 (15.2,37.3) | 1469 (897,2041) |
| S1C,D | 1 mg/kg Cry5B | 27.8 (22.5,33.0) | 1844 (1510,2177) |
| S1C,D | 0.33 mg/kg TrBD | 28.0 (20.3,35.7) | 1163 (932,1393) |
| S1C,D | 1 mg/kg TrBD | 23.3 (10.4,36.1) | 1425 (-132,2982) |
| S1C,D | 3 mg/kg TrBD | 10.3 (3.3,17.2) | 269 (-35,572) |
| S1C,D | 9 mg/kg TrBD | 2.0 (0.7,3.3) | 31.3 (-68,131) |
| S1C,D | 0.33 mg/kg Cry5B  + 0.33 mg/kg TrBD | 16.3 (11.7,20.8) | 1069 (755,1383) |
| S1C,D | 0.33 mg/kg Cry5B  + 1 mg/kg TrBD | 24.8 (8.7,40.8) | 1413 (812,2013) |
| S1C,D | 0.33 mg/kg Cry5B  + 3 mg/kg TrBD | 9.5 (2.1,16.9) | 250 (-12,512) |
| S1C,D | 0.33 mg/kg Cry5B  + 9 mg/kg TrBD | 6.3 (-2.6,15.1) | 188 (-159,534) |
| S1C,D | 1 mg/kg Cry5B  + 0.33 mg/kg TrBD | 16.8 (10.0,23.6) | 1044 (-84,2171) |
| S1C,D | 1 mg/kg Cry5B  + 1 mg/kg TrBD | 14.8 (1.0,28.5) | 781 (-37,1600) |
| S1C,D | 1 mg/kg Cry5B  + 3 mg/kg TrBD | 7.0 (-8.4,22.4) | 325 (-162,812) |
| S1C,D | 1 mg/kg Cry5B  + 9 mg/kg TrBD | 2.8 (-2.3,7.8) | 50 (-84,184) |

^a^ Average hookworm burdens (95% confidence limit).

^b^ Average fecal egg counts burdens (95% confidence limit).
